# *Nocardia veterana* endogenous endophthalmitis in a cardiac transplant patient

**DOI:** 10.1186/1869-5760-3-44

**Published:** 2013-03-30

**Authors:** Megan Scott, Sonia Mehta, Hassan T Rahman, Hans E Grossniklaus, Steven Yeh

**Affiliations:** 1 Division of Vitreoretinal Surgery and Disease, Department of Ophthalmology, Emory University School of Medicine, Atlanta, GA, USA; 2 Uveitis and Ocular Immunology Service, Department of Ophthalmology, Emory Eye Center, Emory University School of Medicine, Atlanta, GA, USA

**Keywords:** *Nocardia veterana*, *Nocardia* species, Endogenous endophthalmitis, Uveitis, Immunosuppression

## Abstract

**Background:**

Endogenous endophthalmitis secondary to *Nocardia* species is extremely rare but often portends a poor visual prognosis often owing to the advanced nature of disease at presentation and delay in diagnosis. Patients who are systemically immunosuppressed are at greatest risk and early suspicion of the role of this organism in patients with acute panuveitis is paramount.

**Findings:**

A 66-year-old cardiac transplant patient on oral prednisone, Myfortic, and tacrolimus developed unilateral panuveitis with a focal white subretinal and retinal lesion. His past medical history was notable for *Aspergillus* pneumonia and cytomegalovirus retinitis in the contralateral eye 12 months prior. Aqueous humor sampling for eubacterial, eufungal, and viral PCR testing, as well as vitreous cultures for bacteria and fungi were unsuccessful in the identification of a causative organism. Progressive enlargement of the lesion was noted despite intravitreal foscarnet, vancomycin, ceftazidime, and voriconazole. A pars plana vitrectomy and retinal and subretinal biopsy led to the identification of *Nocardia veterana*, a recently identified *Nocardia* species. A combination of linezolid, meropenem, azithromycin, ceftriaxone, and intravitreal amikacin resulted in eradication of the infection.

**Conclusion:**

This is the first reported case of *N. veterana* endogenous endophthalmitis in an immunosuppressed patient. Pars plana vitrectomy with a subretinal biopsy was required to establish a diagnosis, as other testing including aqueous PCR and vitreous cultures were negative. The poor visual outcome in our patient highlights the importance of early consideration of *Nocardia* in the differential diagnosis of panuveitis with subretinal disease in the context of immunosuppression.

## Findings

### Introduction

*Nocardia* species are gram-positive, aerobic, filamentous rods found in soil and vegetation, which may cause opportunistic infections in immunocompromised individuals. Most commonly associated with localized pulmonary infections, nocardiosis has also been reported in association with cerebral and pulmonary invasion, and life-threatening systemic dissemination. Recently, polymerase chain reaction (PCR) testing has led to the identification of a novel *Nocardia* species, *Nocardia veterana*[1]. Although there are rare case reports of *N. veterana* infection of the lungs, abdomen, and disseminated disease [2,3], there are no prior reports of *N. veterana* as a cause of endogenous endophthalmitis. We report a case of *N. veterana* endogenous endophthalmitis in an immunocompromised host following cardiac transplantation. Pars plana vitrectomy and subretinal biopsy were required to establish the diagnosis and initiate appropriate antimicrobial therapy.

### Case report

A 66-year-old male presented with decreased vision, floaters, and mild pain in the right eye (OD) of 1-week duration. Past medical history was significant for orthotopic cardiac transplant 18 months prior, diabetes, and *Aspergillus* pneumonia 2-months prior. The patient had a history cytomegalovirus (CMV) retinitis in the left eye for which he received a ganciclovir implant and which we observed to be inactive for 1 year. His medications included prednisone 20 mg daily, mycophenolic acid (Myfortic, Novartis Pharmaceuticals Corporation, Summit, NJ, USA) 180 mg twice daily, tacrolimus (Prograf, Astellas Pharma US, Inc., Northbrook, USA) 1.5 mg daily, and posaconazole 20 mg daily. Ophthalmic drops included brimonidine 0.15% three times daily, and prednisolone acetate 1% (Pred Forte, Allergan, Inc., Irvine, USA) once daily in the left eye.

**Figure 1:**
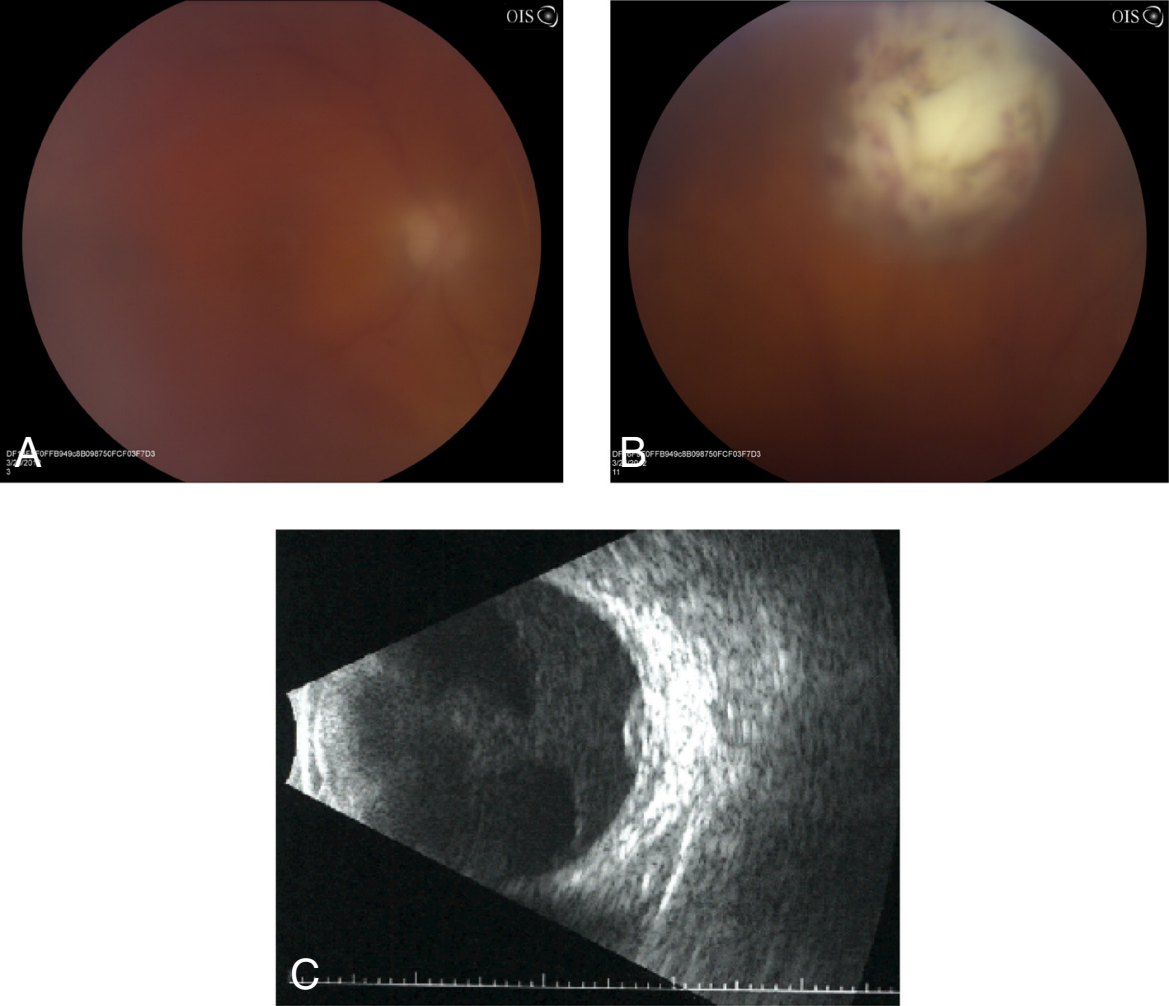
**Photograph and B-scan ultrasound of the left eye.** (**A**) Fundus photograph of the left eye at presentation showing 2+ vitreous haze obscuring the posterior pole. (**B**) At the 12:30 meridian, there is a dome-shaped region of retinal and subretinal whitening with overlying punctate retinal hemorrhage. (**C**) B-scan ultrasound confirming the presence of a superior subretinal mass measuring 1.7 mm in height and 7.1 × 7.2 mm in basal diameter.

Ophthalmic examination showed visual acuities of counting fingers at 2 feet in the right eye and 20/20 in the left eye. His pupils were briskly reactive without an afferent pupillary defect. The intraocular pressures were 12 mmHg OD and 13 mmHg OS. Slit lamp examination of the right eye revealed trace conjunctival injection with 2 to 3+ anterior chamber and vitreous cell. Slit lamp examination of the left eye was unremarkable. Fundus examination of the right eye showed 2+ vitreous haze with a poorly visualized white retinal and subretinal lesion in the superior periphery with overlying punctate hemorrhages (Figure 1). Fundus examination of the left eye showed a chorioretinal scar superotemporally corresponding to the area of prior CMV retinitis. There was no vitreous haze or active retinitis, and a ganciclovir implant was well-positioned inferotemporally.

The differential diagnosis included CMV retinitis, endophthalmitis from fungus, bacteria, mycobacteria, or atypical mycobacteria, toxoplasmosis, EBV-related lymphoproliferative disorder, and intraocular lymphoma. Given the patient's history of CMV retinitis in the fellow eye, the patient underwent anterior chamber paracentesis and intravitreal foscarnet injection (2.4 mg/0.1 mL). Aqueous PCR testing for *Varicella zoster* virus (VZV), CMV, *Herpes simplex* virus (HSV), and *Toxoplasma gondii* DNA were negative. Bacterial and fungal blood cultures showed no growth, and *T. gondii* IgG and IgM were negative. Valganciclovir therapy was deferred because of baseline neutropenia. Prednisolone acetate 1% every 2 h and atropine sulfate 1% were initiated with some improvement of the patient's photophobia.

**Figure 2:**
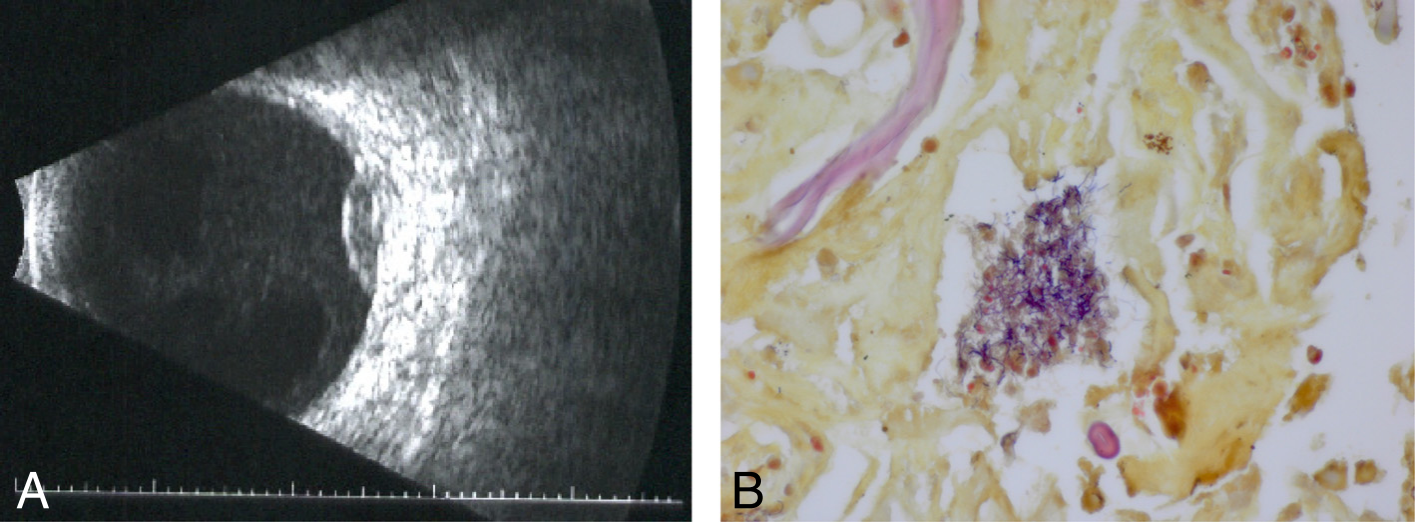
**Repeat B-scan ultrasound and pathologic evaluation.** (**A**) Repeat B-scan ultrasound 1 week following initial presentation confirmed enlargement of the lesion measuring 2.6 mm in height and 9.7 × 10.3 mm in basal diameter. (**B**) Pathologic evaluation of the subretinal aspirate and vitreous specimen showed a cluster of gram-positive filamentous organisms consistent with *Nocardia* species (Brown and Brenn method, ×100).

One week later, the patient's visual acuity decreased to hand motions. The patient continued to have 2+ anterior chamber cell and 3+ vitreous haze, and the area of the retinal and subretinal whitening had expanded (Figure 2). Intravitreal injections of vancomycin (1 mg/0.1 cc), ceftazidime (2.25 mg/0.1 cc), and voriconazole (400 mcg/0.1 cc) were administered. Vitreous cultures were sent for gram and fungal stains, as well as bacterial and fungal cultures. Aqueous fluid was sent for eubacterial PCR, eufungal PCR, and *Aspergillus* PCR, given the patient's recent history of *Aspergillus* pneumonia. All tests were negative.

The patient subsequently underwent diagnostic pars plana vitrectomy and a retinal biopsy, given the enlargement of the subretinal mass and unknown etiology. During the vitrectomy, a few retinal holes were observed in the region of the subretinal mass. These holes were used to aspirate the subretinal material. The vitreous specimen, subretinal aspirate, and retinal biopsy were sent for pathologic evaluation. Air-fluid exchange, endolaser, and 14% C_3_F_8_ gas tamponade were performed, and intravitreal ceftazidime (2.25 mg/0.1 cc) and vancomycin (1 mg/0.1 cc) were administered. Specimens were sent for flow cytometry; PCR testing for VZV, HSV, CMV, and toxoplasmosis; and for gram stain, AFB stain, aerobic, anaerobic, and fungal cultures. Pathologic evaluation of the subretinal aspirate and vitreous specimen showed gram-positive filamentous bacteria consistent with *Nocardia* or *Actinomyces*. Cultures continued to show no growth, and the samples were sent to the molecular diagnostics laboratory of the Centers for Disease Control (Atlanta, Georgia, USA) for 16S ribosomal RNA analysis. The vitreous fluid culture eventually grew *N. veterana* identified using PCR. The specimen was sensitive to amikacin, clarithromycin, linezolid, and trimethoprim/sulfamethoxazole with intermediate sensitivity to ceftriaxone and imipenem.

The patient was admitted to the hospital for intravenous (IV) meropenem (2 g loading, followed by 1 g IV every 12 h adjusted for creatinine clearance of 29 mL/min) and linezolid (600 mg IV every 12 h). Aztihromycin 500 mg daily was substituted for linezolid after 6 weeks because of thrombocytopenia. In addition, meropenem was discontinued after 6 weeks and switched to ceftriaxone 2 grams IV every 12 h for more reliable central nervous system penetration. Both azithromycin and ceftriaxone were continued with a plan to continue the medications for 12 months. Magnetic resonance imaging showed no evidence of brain involvement. On ophthalmic exam, the retinal biopsy site was flat, and the subretinal infiltrates appeared stable. In conjunction with systemic antibiotics, intravitreal amikacin (400 mcg/0.1 cc) was administered weekly for 3 weeks with continued improvement of the retinal and subretinal lesion.

Two months following vitrectomy, the patient's visual acuity declined to light perception only, and a retinal detachment was observed on B-scan ultrasound. The patient underwent vitrectomy, membrane peel, and silicone oil tamponade with successful reattachment of the retina. His visual acuity improved to counting fingers at 9 months follow-up with an attached retina and no evidence of recurrent infection.

### Discussion

Ocular infection by *Nocardia* is rare and most cases are acquired exogenously from trauma or following ocular surgery. *Nocardia* endogenous endophthalmitis is exceedingly rare, with fewer than forty cases reported since 1967 [4]. The majority of these cases have been reported in immunocompromised patients as a result of hematogenous dissemination, most often from a primary pulmonary focus. Associated causes of immunosuppression in these individuals have included untreated HIV infection, organ transplantation, systemic lupus erythematosus, cancer, and steroid use [3]. Our case was unique in that *N. veterana* has not been previously reported to cause endophthalmitis.

*N. veterana* was first isolated in 2001 from a veteran soldier in Australia with bilateral upper lobe pneumonia, giving rise to its *veterana* nomenclature and bears 16S rDNA similarities to *N. vaccinii*[1]. Notably, several cases of *N. veterana* lung infection have also been associated with concurrent aspergillus, CMV, or *Pneumocystis jiroveci* infection [3,5-7], likely as a result of severe immunosuppressed status of the host. Our patient presented with a history of CMV retinitis and *Aspergillus* pneumonia, which preceded the development of *N. veterana* endophthalmitis. A decrease in the immunosuppressive medications and concomitant anti-microbial therapy eventually led to eradication of the intraocular infection. Although the patient's visual acuity was poor at final follow-up, the retina remained attached and the globe was salvaged in this case.

Prior studies have estimated that 0.6% to 1% of patients with systemic nocardiosis will develop endogenous endophthalmitis [4]. Only two isolated infections have been reported in the absence of systemic infection. The diagnosis is often difficult to establish because of its slow growth on culture media and lack of definitive clinical findings. Moreover, diagnostic and therapeutic guidelines for ocular infection have not been established and are frequently associated with poor visual and anatomic outcomes [1].

In a review of 38 patients with endogenous *Nocardia* endophthalmitis reported over a 40-year period, the majority of patients had underlying systemic illness resulting in immunosuppression; specifically, 46% of those affected were transplant recipients, 24% had autoimmune disease, and 19% had hematologic malignancy. In those cases of endophthalmitis associated with transplantation, nocardial infection occurred within the first year of transplantation in 11 of 17 cases. Corticosteroids were the most common immunosuppressive therapy used by 73% alone or in combination with other immunosuppressive medications [4]. Our patient's nocardial endophthalmitis occurred 18 months after transplantation while on combination immunosuppression with prednisone 20 mg/day, mycophenolic acid, and tacrolimus.

The most typical presentation of nocardial endophthalmitis is decreased vision and eye pain. The most common examination findings are the presence of anterior chamber inflammation (37%), vitreous inflammation (37%), and a single chorioretinal lesion (69%). Diagnosis in most cases required diagnostic vitrectomy for ocular specimens (50%) with an average time to diagnosis of 3.5 weeks [4].

Retinal detachment has been reported in up to 40% of patients with nocardial endophthalmitis [4]. Our patient experienced a retinal detachment related to proliferative vitreoretinopathy, which ultimately required repeat vitrectomy, membrane peeling, and long-term silicone oil tamponade. An improvement in the nocardial infection was observed at the time of the retinal detachment repair, although long-term antibiotics are being continued to ensure disease control. The strain of *N. veterana* in our patient was susceptible to multiple antibiotics, and prior reports suggest that the majority of such patients may be treated successfully with trimethoprim and sulfamethoxazole. However, 13% of nocardial endophthalmitis patients were not effectively treated using this trimethoprim/sulfamethoxazole alone [4]. For this reason, intravitreal amikacin has been reported as an alternative treatment strategy and was used in combination with systemic antimicrobials to achieve control of the nocardial infection.

The systemic and visual prognosis for patients with *Nocardia* endogenous endophthalmitis is extremely guarded to poor in many cases, often because of delayed diagnosis and other sequelae including the high frequency of secondary complications, such as retinal detachment, that may lead to visual loss. Specifically, in one series, 32% of patients died, only 31% of patients recovered visual acuity of 20/40 to 20/200, and 34% of patients were 20/200 or poorer; moreover, 31% of patients were 20/40 or better at final follow-up [4].

In summary, this is the first report of endogenous endophthalmitis from *N. veterana*. Although our patient's infection was eventually controlled with antimicrobial treatment, his visual acuity was ultimately limited by the retinal detachment, likely related to the severe, advanced nature of the infection and development of proliferative vitreoretinopathy. *Nocardia*, while a rare cause of endophthalmitis, should be considered along with other opportunistic infections in patients with severe immunosuppression presenting with endophthalmitis.

### Consent

Written informed consent was obtained from the patient for publication of this report and any accompanying images.

## Competing interests

The authors declare that they have no competing interests.

## Authors’ contributions

MS, SM, HTR, HEG, and SY collected, managed, analyzed, and interpreted the data. All authors read and approved the final manuscript.
